# Investigating smart city adoption from the citizen’s insights: empirical evidence from the Jordan context

**DOI:** 10.7717/peerj-cs.1289

**Published:** 2023-03-20

**Authors:** Muneer Nusir, Mohammad Alshirah, Rayeh Alghsoon

**Affiliations:** 1 Department of Information Systems/College of Computer Engineering and Sciences, Prince Sattam bin Abdulaziz University, Alkharj, Riyadh, Saudi Arabia; 2 Information Systems Department, Al al-Bayt University, Mafraq, Jordan; 3 Computer Engineering Department, Al-Ahliyya Amman University, Amman, Jordan

**Keywords:** User acceptance, Technology acceptance model, Smart city, Adoption, Jordan

## Abstract

This study aims to investigate the factors that perceive citizens’ intention to adopt smart city technologies in the Arab world. A self-administered questionnaire that included 312 end users as citizens in Amman, Jordan’s capital city, was used in this study. This study uses advanced statistical techniques to test an expanded technology acceptance model (TAM) that incorporates the determinants of perceived usefulness, perceived ease of use, security and privacy, ICT infrastructure and inadequate Internet connectivity, social influence, and demographic profiles. Based on the results, perceived ease of use and ICT infrastructure and Internet connectivity showed positive association with the intention of citizens to adopt smart city services in Jordan. By recognizing the factors that predict citizens’ adoption of smart city services, this study presents some theoretical implications and practical consequences related to smart city service adoption.

## Introduction

The term smart city has been used and repeated in numerous studies since 2010, covering different embodiments of the city, comprising of ‘intelligent city,’ ‘the digital city,’ ‘the sustainable city,’ ‘the ubiquitous city,’ and ‘the knowledge city’ (Han & Kim, 2021). However, the diverse meanings of the concept of a smart city and digital city among the most frequently used terms to identify and define the technological and smart capability of a city in the plurality of research studies. However, these smart capabilities and technologies have not been applied in accordance with a particular meaning (Cocchia, 2014). In addition, sustainable projects of development for adopting smart cities in developing countries have faced boundaries and challenges (Vu & Hartley, 2018; Tan & Taeihagh, 2020). Hence, this study focuses on adopting smart cities in developing countries, that is undertaking a study to investigate and identify these challenges and the possible opportunities of enabled 5G technologies. This contributes to exploring the various aspects of rapid urbanization and the changes that can be accommodated through the use and activation of these technologies.

Previous studies on smart city adoption have focused on technology adoption and engagement (Granier & Kudo, 2016; Han & Kim, 2021), ignoring the revolution of information and communication technology (ICT). Thus, the factors studied in this study may be beneficial in investigating adoption and acceptance from user intention of smart city technologies, as rigorous empirical evidence studies of smart city adoption from the intention behavior of citizens in the Arab nation is still yet to engender (Aina, 2017; Shareeda, Al-Hashimi & Hamdan, 2021). This study is among the leading empirical evidence of studies that concern citizens’ behavioral intention towards smart cities’ concept of adoption in Jordan. Jordan has made very slow progress in the domain of development projects of smart cities recently (Bazazo, Alananzeh & Alrefaie, 2022), which has substantial implications for managerial staff, policymakers, and researchers in the region. In addition, further study on the adoption and engagement frameworks of smart cities is rigored cross cultures. Thus, investigating the factors that lead to adoption and acceptance of smart city development in Amman, Jordan’s capital city is crucial to understand the potential of smart technologies in developing countries, particularly in the Arab world (*i.e*., Jordan context).

This study makes numerous significant empirical and theoretical contributions to studies on IT. First, it utilizes the technology acceptance model (TAM) and the unified theory of acceptance and use of technology (UTAUT) model to explore and investigate end-users’ (Jordanian citizens) decisions to adopt and accept smart cities in a developing country (Jordan as a case study), which has not been broadly explored and mentioned in previous studies. Second, we add five constructs–perceived usefulness, perceived ease of use, security and privacy, ICT infrastructure, and inadequate Internet connectivity and social influence–into the TAM model, including demographic profiles, to obtain a better realization of the role of these diverse items on citizens’ behavioral intentions to adopt and accept smart cities. Third, this study contributes to expanding the knowledge and science in this area by investigating whether the effect of extended TAM on smart cities might be moderated by gender, age, monthly household income, ICT knowledge, and education of citizens as end users. Finally, the results of this study will influence decision makers concerned with boosting and stimulating smart city technologies among Jordanian people. The prime objective of this study is to explore user adoption and acceptance by citizens in Amman, Jordan’s capital city, based on their current experience with smart technologies delivered in their lives. To achieve this, we conducted a literature review of smart city development, built a proposed research model based on the extended TAM model and hypotheses development, and posed the following research questions: (i) what are the key factors contributing to the failure or hindrances of adoption of smart city technologies and development in developing countries? (ii) Are there barriers to the acceptance of technologies of smart city that can be brought to citizens in developing countries based on citizens’ perspectives?

The following sections of this paper are organized as follows. The next section reviews the relevant literature. This is followed by the development of the hypotheses and this paper proposed model. A section on the research methods is then presented. The results of this study are then presented and discussed. Finally, the paper outlines contributions, limitations, and future work for further study in the final section.

## Research Background

### Smart city adoption and development in Amman, Jordan’s capital city

The Greater Amman Municipality plan to transform Amman into a smart city starts in 2023. By reviewing the literature, it becomes clear that Jordan is still a novice in the field of smart cities; there are very few studies on smart city development in Jordan (Shaqrah, 2019; Fernandez-Anez et al., 2020). Some researchers have proposed smart projects that can fit the smart city if employed. Researchers (Tahat et al., 2018; Shaqrah, 2019) presented an environmental project to perform real-time measurements onboard a moving vehicle. However, no study has addressed the main challenges of readiness.

**Figure 1:**
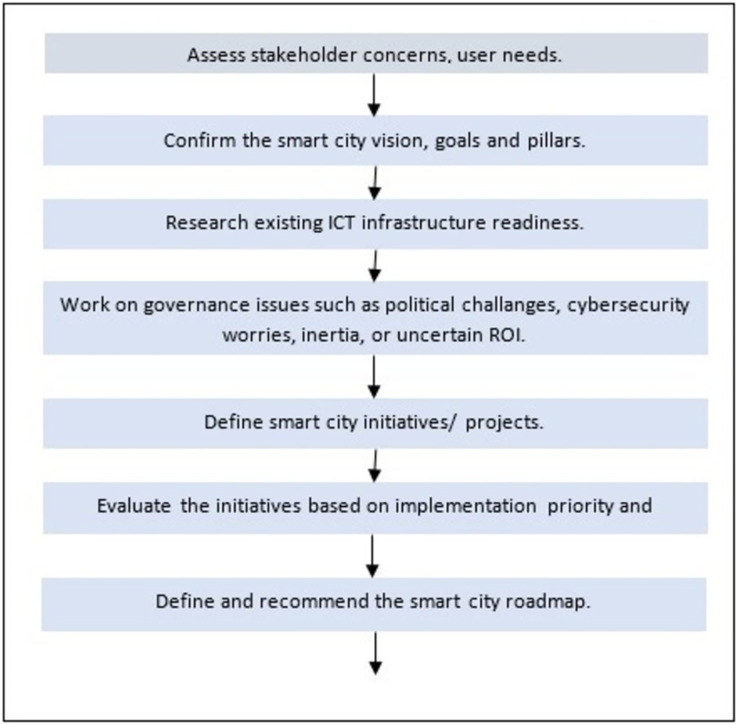
Amman’s smart city roadmap (cited from Modee Government Jordan (2021)).

The Greater Amman Municipality, which is the official authority responsible for implementing the smart city, has published a roadmap to the smart city following a step-by-step approach, as shown in Fig. 1. The first step is to estimate needs and available capabilities, then decide the required technical issues, and finally define the smart city.

In terms of readiness, the roadmap explicates the faces of the challenges to achieve this vision. The main reported challenges are population growth, growing traffic congestion, human resource capabilities for smart solutions, and global and regional economic challenges.

The vision of this smart city includes five pillars: smart mobility, energy, environment, public safety, and public health. A smart city should be implemented in two phases, each containing a number of projects that satisfy these pillars. The key enablers of these projects include infrastructure and data analytics, governance and regulation, outreach, funding, financing, and talent/human resources.

### Factors affecting the adoption of smart cities: technology acceptance model (TAM)

Various studies have recommended and observed several models to investigate the key determinants of adoption and development of information technology (IT). The TAM is considered one of the best models proposed in the IT adoption literature (Davis, 1989). Perceived usefulness and ease of use are the two main factors that influenced the original TAM (Davis, 1989).

#### Perceived usefulness and enjoyment

The acceptance and participation of the population is the most important factor in the success of technology initiatives (Li et al., 2018). It can be considered that the article of “Perceived Usefulness, Perceived ease of Use, and User Acceptance of Information Technology” (Davis, 1989) is the basis of the technology acceptance models. Perceived usefulness can be defined as the belief of people that a particular system will improve their work performance. The acceptance and interaction of citizens with ICT and Internet of Things (IoT) have been examined by many studies from different countries to study the possibility of moving to the stage of smart cities. Chaiyasoonthorn, Khalid & Chaveesuk (2019) examined the importance of perceived usefulness on retention behavior in Thailand. Lin et al. (2019) and Zavratnik et al. (2020) addressed the development of smart cities by considering the well-being of residents from various aspects. Therefore, we propose the following hypothesis:

HC1: Perceived usefulness has a positive relationship with Jordanian users’ intentions to adopt smart city services.

#### Perceived ease of use

According to Davis (1989), perceived ease of use is defined as less effort required by people to use a new system or technology. It is observed that the ease of use is one of the key issues regarding the adoption of smart cities (Ismagilova et al., 2019). Lytras & Visvizi (2018) attempted to answer the question of who uses smart cities and what are their reservations regarding the ease and efficiency of its use. They identified that even the most educated users expressed serious concerns regarding ease-of-use issues. It is emphasized that the complexity of a particular system would become a disincentive that discourages the adoption of any innovation (Ooi et al., 2011). Mohamudally & Armoogum (2019) studied the factors that affected different items regarding smart cities in Mauritius, including perceived ease of use, and inferred the same results. Thus, we propose the following hypothesis:

HC2: Perceived ease of use has a positive relationship with Jordanian users’ intention to adopt smart city services.

### The extended model of TAM and the hypotheses development

#### Security and privacy

Security is a dynamic concept that includes an attempt to prevent direct or indirect physiological or digital harm that affects the life of the smart city population. Braun et al. (2018) discussed the main five security challenges in smart cities: data sharing and mining, mashup data, cloud security, secondary use of collected data, and threats of artificial intelligence. The main challenge is to ensure interoperability between devices while maintaining secure and private services (Ejaz & Anpalagan, 2019). A smart city consists of four layers: perception, network, service, and application. Each layer has its own security issues and the proposed solutions. For instance, the perception layer suffers from DoS and routing attacks, whereas the network layer suffers from various attacks owing to the diversity of its protocols. The service layer relies on cloud computing, which requires secure processing to maintain scalability, availability, and immutability. Rao & Deebak (2022) reviewed different threats and techniques to address the main security challenges in smart cities. Thus, we propose the following hypothesis:

HC3: Security and privacy have a positive relationship with Jordanian users’ intentions to adopt smart city services.

#### Social influence

Social influence can be defined as the extent to which an individual perceives that significant others think the person should use the new technology (Chang, Wang & Wills, 2020). In the initial adoption stage, opinions expressed by reference groups have greatly influenced individual’s beliefs. Reference groups include the opinions of experts, close friends, and family, in addition to mass media such as the Internet and TV. Studies prove that simply knowing that others are using an innovation is enough to motivate a person to use and adopt it (Schepers & Wetzels, 2007). Studies in social psychology also indicate that being aware that others are performing a particular behavior can directly influence our behavior (Chatterjee, Sarker & Valacich, 2015; Kulviwat, Bruner & Al-Shuridah, 2009; Talukder & Quazi, 2011). Studies have also shown that people tend to join groups that are similar to themselves, and the more they are in a group, the more they participate in what others participate (Hafer & Ran, 2016). Therefore, we propose the following hypothesis:

HC4: Social influence has a positive relationship with Jordanian users’ intention to adopt smart city services.

#### ICT infrastructure and inadequate Internet connectivity

Smart infrastructure has become an essential solution for urbanization problems, which requires to employ ICT to be more effective and sustainable (Serrano, 2018). Balakrishna (2012) analyzed the potential of smart mobile devices based on embedded sensors. Three main smart infrastructure indicators were proposed: awareness of the real world by capturing and analyzing big data, knowledge engineering that generates exploitable knowledge from big data, and interconnection, which proposes a network for data-driven knowledge exchange across all areas of the city. This can be compared to the concept of urban metabolism, which suggests the strict control and monitoring of city inputs to achieve faster sustainability outcomes (Allan, 2020). Kuberkar & Singhal (2020) identified that the existence of conductive infrastructure was necessary for commuters to use a public transport chatbot within a smart city (Costales, 2022). Different studies and theories discussing infrastructure regarding smart transformation and smart cities are entailed in study by Dirsehan & van Zoonen (2022). Thus, we propose the following hypothesis:

HC5: ICT infrastructure and Internet connectivity have a positive relationship with Jordanian users’ intentions to adopt smart city services.

#### Demographic variables

The determination of users’ acceptance of technology is an ongoing administrative challenge owing to the diversity of technology forms, uses, and applications (Schwarz, 2007). The unified theory of UTAUT (Venkatesh, 2003) has listed a group of constructs and moderators such that researchers can assess the willingness and intention of the targeted class towards the proposed system. Constructs included effort expectancy, social influence, facilitating conditions, and performance expectancy. In contrast, the moderators included experience, age, and gender.

UTAUT was developed for the organizational context to determine the user’s behavioral intention within the same organization. However, the constructs and moderators inside one organization are limited to wider environments that may have several types of tasks and more complex interactions (Brown, 2006). Therefore, UTAUT2 (Venkatesh, Thong & Xu, 2012) was developed based on the UTAUT. UTAUT2 adds three new determinants which include: habit, price value, and hedonic motivation. However, UTAUT2 still suffers from some limitations in determining the acceptability of emerging technologies with more complex characteristics. In this study, we considered five demographic characteristics: gender, age, education level, monthly income, and ICT experience.

Gender challenges in the attitude toward computers and the Internet have been addressed in several studies. For instance, a difference was observed in the feelings of students of the two genders toward computers (Qureshi & Hoppel, 1995). Moreover, differences were observed in computer skills, where males appeared to have better skills than females (Harrison & Rainer, 1992). However, studies have shown that women are more amenable to technostress and computer anxiety than men (Igbaria & Chakrabarti, 1990). Recently, it was observed that boys have more interest in computers than girls during adolescence, but these changes occur in the advanced age stages (Lee et al., 2019). On the other hand, males showed better computer efficacy and comfort than females.

In the case of Jordan, Aljaraideh & Al Bataineh (2019) studied the barriers to utilize online learning in Jordan and observed that females tended to face more barriers than males. In an interesting cross-national study by Ameen, Willis & Shah (2018) addressed the gap between the use and adoption of smartphones in the UAE and Jordan. The results showed that only Jordanian men realized the usefulness of their smartphones and mobile applications. Although both genders in the UAE were aware of this point, however females showed lesser awareness than males. This is attributed to the fact that Jordanian women use less smart devices and are therefore less aware of their usefulness. In terms of ease of use, the results explained that there were no major differences between the two countries, where all participants agreed that the effort needed to use smartphones is greater, except for Jordanian males. Regarding enjoyment, while females use smartphones less than males, they achieved similar results to males in both countries. Females place great emphasis on the pleasure associated with their use. In an unexpected result, the influence of the Arab culture that favors face-to-face meetings is unimportant to Emirati men because they are the highest users of smart devices in the Arab world. In terms of the habit effect on the behavioral intention of consumers, the results showed that, in contrast to the UAE, there are no significant differences in Jordan.

With the rapid growth of people in developed countries, it is important to care about the quality of electronic services used in daily life to preserve society from segregation by age and to ensure a better life, particularly for the elderly. Although the skills of older people in using technology are improving over time (Šimonová et al., 2017; Thomas et al., 2021; Álvarez-Dardet, Lara & Pérez-Padilla, 2020), the age gap is still an important factor in the use of electronic services and may cause digital inequality (Wagner, Hassanein & Head, 2010). Having age as a moderator increases the explanatory power of TAM. The age of consumers is particularly useful in explaining the differences in smart city adoption behavior (Chung et al., 2010).

In a large and diverse sample, Lee et al. (2019) indicated that there were differences in attitudes toward computers based on age, where older adults reported less comfort and effectiveness in using computers than younger adults. Meanwhile, there is a group effect (year of birth). In general, attitudes are more positive among recent birth cohorts.

Popova & Zagulova (2022) asserted that the use of technology gap increases with age, and therefore, there was a need to continue digital literacy to implement smart cities like Australia (Thomas et al., 2021). On the contrary, age was observed to have a negative impact on the relationship between performance expectation and facilitating condition owing to its influence on device availability and knowledge. According to Worldometer (https://www.worldometers.info/world-population/jordan-population/), the median age of Jordanians is 23.8 y. Meanwhile, Al-Jamal & Abu-Shanab (2015) observed that older Jordanian citizens have less intentions to use e-government than younger citizens.

In Jordan, the literacy rate is approximately 98.23%, as asserted by different resources (Mahasneh et al., 2021). Most of the time, educational level has not been examined as a primary construct in data analysis. Instead, it is usually measured as a demographic characteristic or control variable. Moreover, researchers generally associate educational level with indirect use through computer anxiety. For instance, Igbaria (1993) reported that educational level has positive and negative effects on perceived usefulness and computer anxiety, respectively. Behavioral intention and perceived usefulness were negatively affected by computer anxiety. In addition, behavioral intentions, attitudes, and user acceptance were positively affected by perceived usefulness.

According to Tsai et al. (2020), more attitudes emerged from those who had more education and experience with computers. Most of the results indicate that higher levels of education are likely to have a positive impact on use. Moreover, a higher level of education can lead to greater computer knowledge, which enables internet use (Lytras & Visvizi, 2018). Based on the study conducted by Vidiasova, Kachurina & Cronemberger (2017), the cities of Bodo, Singapore, Delft, Melbourne, and Toronto attained the highest scores among the 20 smart cities. The results emphasized that education positively affects other components of the smart city.

When great intentions are provided to age and gender in the adoption of smart city, the division of residents into several socioeconomic classes may provide a different view of behavioral intention. Individual income may influence the adoption and use of smart cities. The International Telecommunications Union (Leong, Ping & Muthuveloo, 2017) asserted that e-commerce is more likely to be adopted by younger people in mostly urban areas and those with higher incomes.

Engebretsen (2005) studied the IT acceptance in low-income African countries and observed a high acceptance of technology despite the lack of a preliminary plan. In the case of Jordan, where the unemployment rate is approximately 43.3%, the latest statistics from the Jordanian Department of Statistics (2019–2020) explains that 15.7% of Jordanians suffer from absolute poverty, which is approximately 1.069 million citizens. Meanwhile, the rate of extreme hunger poverty was approximately 0.12%, representing approximately 7,993 individuals.

Leong, Ping & Muthuveloo (2017) studied the adoption of the IoT in a Malaysian smart city. Their focus was on the role of experience as a moderator in the relationship between behavioral intention and determinants. The results indicate a negative effect of experience on the relationship between behavioral intention and perceived security risk, where a person who had more experience in technology was expected to be more aware of security. This is similar to the result of Hsu, Lee & Su (2013), who observed that perceived security was considerably influenced by IT literacy. In addition, the relationship between performance expectancy and behavioral intention was observed to be negatively affected by experience, although Maduku (2015) denied this moderating effect.

Aliyu, Alhassan & Hussaini (2021) observed that experience has a positive moderating effect between behavioral intention and smart perceived trust, where more experienced users are more likely to trust and continue using the system. A past study by Warkentin et al. (2002) revealed that the relationship between behavioral intention and effort expectancy was positively affected by experience, such that more experienced users were looking for ease of use in the adoption of the IoT, which confirmed the results of Mei-Ying, Pei-Yuan & Weng (2012). Finally, a positive effect was observed on the relationship between habit and behavioral intention.

This study proves that the effect will be significant for citizens who have high ICT skills and experience compared to those with low ICT skills and experience. Hence, the following hypothesis is proposed:

HC1a: Gender, age, ICT experience, education, and household income significantly moderate the relationship between perceived usefulness and Jordanian users’ intention to adopt smart city services.

HC2a: Gender, age, ICT experience, education, and household income significantly moderate the relationship between perceived ease of use and Jordanian users’ intention to adopt smart city services.

HC3a: Gender, age, ICT experience, education, and household income significantly moderate the relationship between security and privacy, as well as Jordanian users’ intentions to adopt smart city services.

HC4a: Gender, age, ICT experience, education, and household income significantly moderate the relationship between social influence and Jordanian users’ intention to adopt smart city services.

HC5a: Gender, age, ICT experience, education, and household income significantly moderate the relationship between ICT infrastructure and Internet connectivity, as well as Jordanian users’ intentions to adopt smart city services.

## Research Methodology and Model

**Figure 2:**
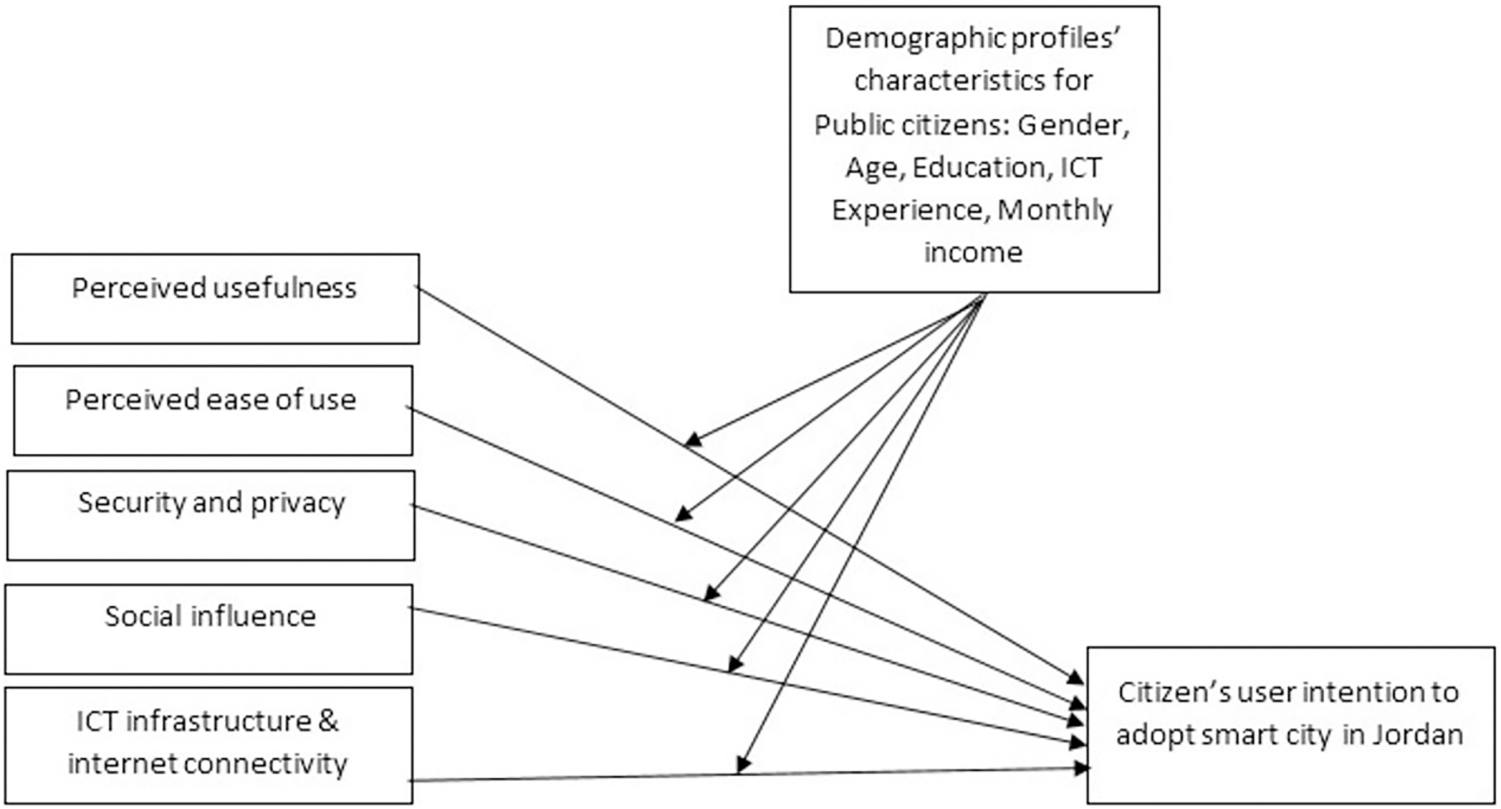
The proposed theoretical model.

After reviewing the literature and based on the developed hypotheses, the proposed theoretical model for this study is shown in Fig. 2. This study proposes that the intention to adopt and accept a smart city is driven by external factors, (*i.e.*, perceived usefulness, perceived ease of use, security and privacy, ICT infrastructure, and inadequate internet connectivity and social influence) with a variety of demographic profiles’ characteristics (See Table 1) as the moderator.

**Table 1:** The socio-demographic characteristics of respondents.

Demographics	Counts	Percentage
Gender		
Male	144	(49.6%)
Female	146	(50.3%)
Age		
18–25 years	60	(21%)
26–35	92	(32%)
36–45	83	(28%)
46–50	34	(12%)
Above 50	21	(7%)
Education		
High school	7	(2.4%)
Diploma degree	14	(4.8%)
Bachelor’s degree	162	(55.9%)
Postgraduate studies	107	(36.9%)
Others	0	(0%)
Information and communication technology (ICT) experience		
Weak	3	(1%)
Acceptable	29	(10%)
Good	59	(20%)
Very good	132	(46%)
Excellent	67	(23%)
Monthly income		
Below 500 JD	74	(26%)
500–700 JD	71	(24%)
700–1,000 JD	67	(23%)
Above 1,000 JD	78	(27%)

### Sample and procedure

Despite the fact that we used the validated measures (See Table 2), a pilot of the questionnaire survey was reviewed by experts (professionals, academics, and decision makers) who led the theoretical and practical development in digital economic and IT to validate that the participant could easily comprehend it and the questionnaires intelligibility aspects from the interviewees’ perspectives. Therefore, the draft was conducted with six seniors who were interested in the smart cities domain; three senior government officers (Amman municipality and Ministry of Digital Economy and Innovation), who are currently employed by government agencies that provide government online services and information and communication technology infrastructure; and three university academic professors whose primary research areas are IT and digital economics. At the end of the pilot stage, the participants explained that the questionnaire was easy to understand and clear. In addition, it was not time-consuming to complete the questionnaire. However, a few minor propositions were provided to enhance and address effectively in the final version of the questionnaire to meet the Jordan’s context.

**Table 2:** Measuring scales and references for the proposed constructs.

Construct	Items	References
Perceived usefulness and enjoyment (four items)	U1: Smart city increases my productivity. U2: Smart city allows me to improve my work performance and quality. U3: Smart city saves me time. U4: Overall, I would find smart cities to be advantageous.	Adapted from: Davis (1989), Ahmad & Khalid (2017), Wang (2014), Hung, Chang & Kuo (2013).
Perceived ease of use (five items)	E1: My interaction with smart city is clear and understandable. E2: Learning to operate smart city and following the guidance is easy to me. E3: I found the smart city is easy to get to do what I wanted to do. E4: It is easy and quick for me to become skillful at using smart city. E5: I found smart city is user friendly and easy to use.	Adapted from: Davis (1989), Ahmad & Khalid (2017); Hung, Chang & Kuo (2013), Wang (2014).
Security and privacy (five items)	P1: My information is not disclosed to unwanted authorities or personals. P2: My confidentialities of information remains protected. P3: The security aspect of the IT-enabled system is not compromised under any circumstances. P4: Users are adequately trained and aware of how to use the IT enabled services safely and securely. P5: Overall, I would find the IT-enabled services of smart city are having a high degree of security features which can keep the digital services fully secured.	Adapted from: Chatterjee, Kar & Gupta (2017), Jnr (2021), Chong (2013).
ICT Infrastructure and Inadequate Internet connectivity (five items)	II1: The IT-enabled services are efficient and user friendly to the residents of smart city. II2: The functionalities are adequately designed to meet the needs of the users with full satisfaction. II3: The systems are well maintained providing good-quality services to the users. II4: The information is continuously updated with latest information in place. II5: The system is reliable and it maintains the performance as per the requirements.	Adapted from: Chatterjee, Kar & Gupta (2017), Borena & Negash (2016), Egoeze et al. (2014), Lin, Fofanah & Liang (2011).
Social Influence (five items)	S1: Friends and family members have influenced my decision to use smart city. S2: Mass media (*e.g.*,: TV, radio and newspaper) have influenced my decisions in using smart city. S3: It is the current trend to use smart city. S4: People whose opinions that I value prefer that I use smart city for doing transaction. S5: I will use smart city if my colleagues use it.	Adapted from: Sim et al. (2014), Ahmad & Khalid (2017), Venkatesh, Thong & Xu (2012).
Behavioural intention (three items)	B1: I will use smart city once adopted. B2: I will purchase smart city enabled phones once adopted. B3: I predict that I will continue to use the smart city applications on a regular basis.	Adapted from: Mital et al. (2018), Yen et al. (2010), Melas et al. (2011).

To empirically test this, 10 hypotheses were developed. The questionnaire survey method was used to develop an online survey, administered as a self-questionnaire using the online questionnaire software Microsoft office/forms (Saunders et al., 2007). A five-point Likert scale ranging from 1 = “strongly disagree” to 5 = “strongly agree” was used to measure all of these independent constructs. Each independent construct was measured using 24 validated items (excluding the demographic variables). English and a professional translation into Arabic were used in the questionnaire writing, as the main language in Jordan is Arabic. The criterion for distributing the questionnaires was based on who had a motivation for participation and, at least novice knowledge about smart city projects. Thus, respondents were able to comprehend the questions in the questionnaire. Furthermore, they will be able to provide the best answers (*i.e*., inputs for adoption and acceptance) for this study. Motivated and willing participants are suitable for this study because their sense of intellectual intention is significant in envisaging and revealing the adoption of smart cities in Jordan.

Random samples of 312 Jordanian citizens from various backgrounds, communities, experiences, education, occupation, and economic background/income were used to reflect the context of Amman, Jordan’s capital city . The survey questionnaires were distributed to various classes with a consent form. Respondents were informed of the purpose of the study. Participants were informed to exclude any data, which led to their profiles to avoid response bias and maintain respondents’ privacy. Despite this sample characteristic, the sample is focused on a younger slice of the Jordanian population without ignoring others, which reveals that they are appropriate as it represents population from diverse contexts in Jordan. Furthermore, they are more highly motivated and willing to accept the concept of adopting advanced or new technologies (smart services in cities) compared to elderly people who do not care about new or advanced technologies. This is compatible with the methods adopted by Chong (2013) and Pan & Jordan-Marsh (2010). They argued that younger people, in general, were more educated and sympathetic to use and adopt information and communication technology. Respondents in the study voluntarily participated using electronic surveys that were specifically constructed to meet the research context formed and purposes. The appropriate sample size is determined based on the number of constructs/variables in research model (Hair et al., 2010); the minimum sample size should be not less than 150 if the number of constructs/factors is seven or less, and each construct has more than three observed items (Hair et al., 2010; Sekaran & Bougie, 2016). Therefore, the sample size (312) in this study was useful and convenient sample based on the proposed research model as shown in Fig. 2. In addition, such ratio of sampled and valid surveys is considered satisfactory (Mellahi & Harris, 2016). However, the questionnaires were initially cleaned and screened, and 22 responses were excluded from the dataset because of high missing values or incorrect answers in the objective manner of this study. The final dataset included 290 valid response questionnaires.

The first model used in this study of smart city acceptance and usage was TAM. This model was proposed in 1985 to ease the acceptance of technological services (Lee, Kozar & Larsen, 2003). Essentially, the model is the incremental development of user motivation to use technology through a stimulus.

This proposed study intends to analyze user adoption and acceptance of smart technologies delivered in their life in Jordan based on their current experience. This study also seeks to test the usefulness of the TAM as a theoretical foundation for insight into the attitudes of users towards the acceptance of smart cities. This study uses statistical techniques to examines an extended (TAM) and the “unified theory of acceptance and use of technology” model (UTUAT) by integrating the factors of usefulness, ease of use, security and privacy, cost and extended payback period, regulatory norms and policies, ICT infrastructure and inadequate internet connectivity, social influence, skilled manpower, operational management and technical knowledge among policymakers, integrity and compatibility, and demographic profiles. Respondents were classified into two groups (citizens and experts). In particular, the number of random citizens in Jordan and number of experts (professionals, academics, and decision makers) who lead the theoretical and practical development of economic digital and IT. The questionnaire was adopted, and the sample was randomly selected from Jordanian citizens.

### Instrument and measurement

The research model (See Fig. 2) is composed of five constructs: perceived ease of use, perceived usefulness, ICT infrastructure and inadequate Internet connectivity, security and privacy, and social influence. Each construct was measured using several parameters. All the measurement parameters were adapted from the literature review to maintain content validity (See Table 2). Table 2 lists the parameters associated with every construct of the proposed model (See Fig. 2) and the literature that was used as a benchmark for their adaptation of both target stakeholders (*i.e*., public citizens). However, a five-point Likert scale is used to measure each item, with one indicating “strongly disagree” and five indicating “strongly agree”.

## Analysis and Results

Given the frequency of the study respondents (290), the socio-demographic shows the characteristics of respondents; where 146 of respondents were females (50.3%), while the remaining 144 were males (49.6%). Regarding age, a majority of the respondents 92 (32%) were aged between 26 and 35. Next, the characteristics of respondents depict the educational distribution of the respondents whereby 162 of respondents (55.9%) were bachelor’s degree, 107 (36.9%) were postgraduate studies, 14 respondents (4.6%) were diploma degree, and lastly seven respondents (2.4%) were high school qualification. Moreover, the largest proportion of respondents (46%) had a very good experience in Information and Communication Technology (ICT). Finally, 78 of respondents had a salary above 1,000 JD.

### Common methods bias testing

The exploratory factor analysis is a group of statistical methods aimed at reducing the number of variables or data related to a specific phenomenon (Watson, 2017). Initially, factor analysis was tested for the appropriateness of the data and its ability to explain the study model. Thus to achieve that, the value of the Kaiser-Meyer Olkin (KMO) test should be between 0.8 and 1 to be an adequate (Shrestha, 2021). The analysis showed that the KMO values for all the constructs are greater than 0.80, which reflects the ability and validity of the data to interpret the proposed model. In addition, the results showed that the initial eigenvalue of Kaiser’s criteria was good and acceptable for all study constructs when compared to the Monte-Carlo simulation Eigenvalue. With regards to the cumulative variance explained, all the constructs had exceeded 70%, which was acceptable (Perito et al., 2020). In addition, the values of Bartlett’s test for homogeneity of variances were statistically acceptable, as the *p*-value was less than 0.000, which reflects the validity and robustness of the statistical analysis (Aslam, 2020).

### Measurement model

In this article, the relationship between the research constructs was examined using Amos in order to test research hypotheses, as it uses multiple methods and patterns that contribute to the completion of statistical analysis on all data (Danks, Sharma & Sarstedt, 2020). Before the application of a questionnaire, confirmatory factor analysis (CFA) was carried out to validate research constructs, where if measurement model has achieved acceptable conformity for quality indicators, it is possible to judge the veracity of constructs and their statements. Therefore, composite reliability (CR), factor loading (λ), Cronbach’s alpha (α), and average variance extracted (AVE) were tested as shown in the Table 3. In this regard, it showed that the Cronbach’s alpha values were high for all research constructs, reflecting the quality and appropriateness of these factors for measuring the research model, as the values approached 0.08 (Mohajan, 2017), as well as AVE are greater than 0.70, which means that the IVs in the tested model are uncorrelated (Valentini & Damasio, 2016). Also, the results showed that values of CR were higher than 0.80, which is acceptable (Purwanto & Sudargini, 2021). Moreover, it is clear that all the constructs and their items have obtained good values, since the factor loading (λ) had exceeded the specified ratio (0.4), which indicates that it is acceptable. Also, many indicators were used to measure the quality of the measurement model and its suitability for the interpretation and testing of the research hypotheses, as these indicators scored high rates, where normed fit index (NFI) reached 0.674, Tucker-Lewis index (TLI) got 0.789, while comparative fit index (CFI) reached 0.824 (Franc et al., 2018; Li, Wang & He, 2018). Moreover, the root mean square error of approximation (RMSEA) was used to find out the percentage of the approximation error, as the percentage was very small (0.061), which means that the model is valid for measurement (Shi et al., 2020).

**Table 3:** Factor loadings, average variance extracted, composite reliability and Cronbach’s alpha.

Demographics	λ	AVE	CR	α
Behavioral intention		0.6	0.8	0.6
B1	0.6			
B2	0.5			
B3	0.7			
Perceived usefulness		0.6	0.7	0.6
U1	0.7			
U2	0.6			
U3	0.6			
U4	0.5			
Perceived ease of use		0.6	0.7	0.6
E1	0.4			
E2	0.6			
E3	0.5			
E4	0.4			
E5	0.5			
Security and privacy		0.5	0.7	0.6
S1	0.6			
S2	0.7			
S3	0.6			
S4	0.4			
S5	0.6			
Social influence		0.6	0.8	0.6
SI1	0.5			
SI2	0.7			
SI3	0.6			
ICT infrastructure		0.6	0.8	0.6
I1	0.6			
I2	0.4			
I3	0.5			
I4	0.5			
I5	0.5			

Regarding the dimensions of the research, namely behavioral intention, perceived usefulness, perceived ease of use, security and privacy, social influence, ICT infrastructure, a discriminant validity matrix was run to ensure that multicollinearity was avoided. Table 4 showed the discriminant validity of the constructs, as it was noted that there was a weak correlation at all, however, in spite of its weakness, the values of discriminant validity were significant at α = 5% (Schwarz, Schwarz & Black, 2014).

**Table 4:** Discriminant validity of the constructs.

		1	2	3	4	5	6
1	Behavioural intention						
2	Perceived usefulness	0.4*(0.000)	1				
3	Perceived ease of use	0.2*(0.003)	0.2*(0.000)	1			
4	Security and privacy	0.3*(0.000)	0.3*(0.000)	0.4*(0.000)	1		
5	Social influence	0.1*(0.003)	0.1*(0.004)	0.1*(0.001)	0.2*(0.001)	1	
6	ICT infrastructure	0.3*(0.000)	0.3*(0.000)	0.2*(0.001)	0.4*(0.000)	0.2*(0.001)	1

**Note:**

* *p* < 0.05; (r2).

### Hypothesis testing

**Figure 3:**
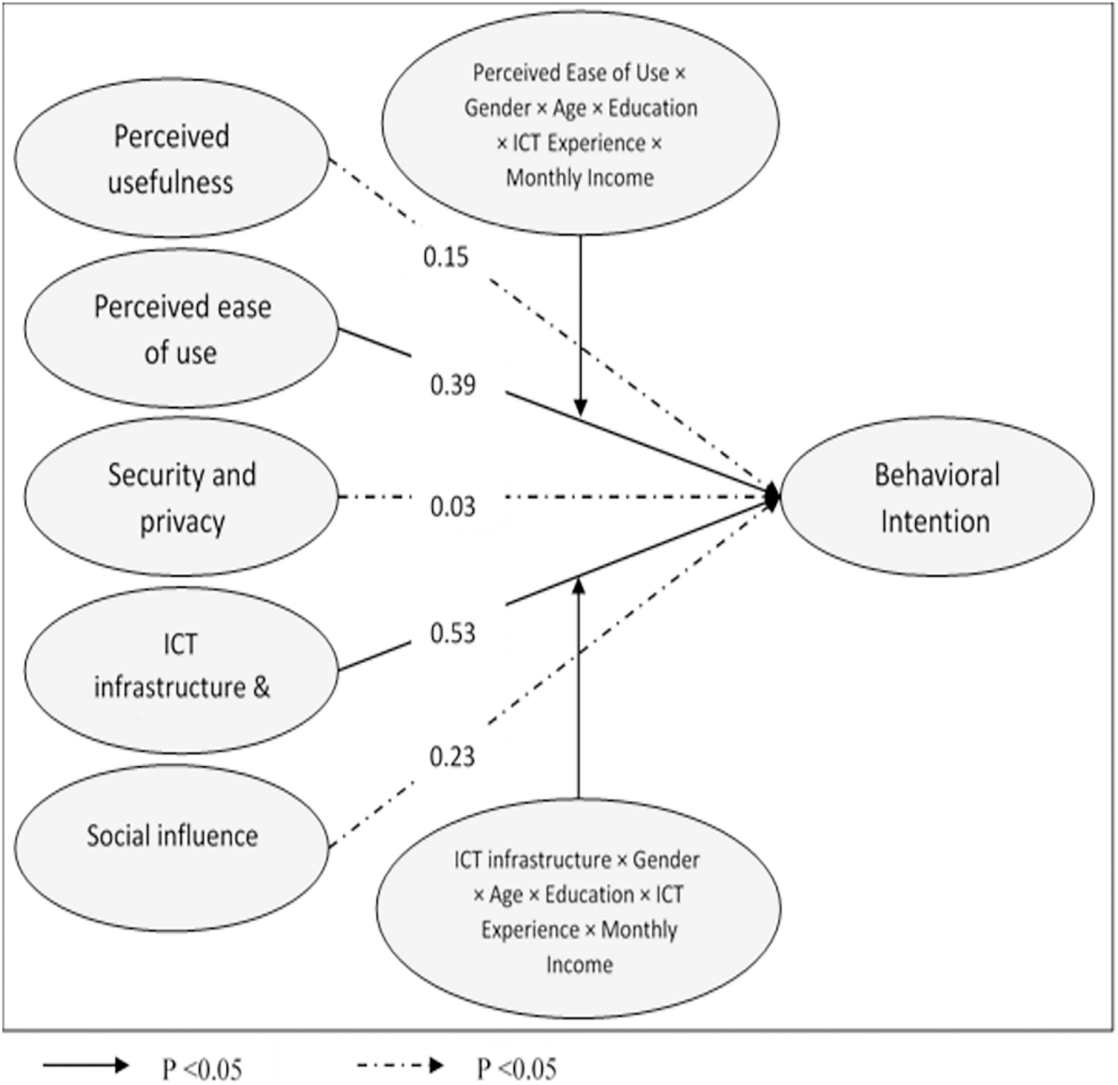
The proposed structure model.

From Table 5, it could be concluded that there was a positive relationship between perceived ease of use and behavioural intention (ß = 0.38; *p* < 0.05, R^2^ = 0.35), social influence and behavioural intention (ß = 0.21; *p* < 0.05, R^2^ = 0.35), as well as ICT infrastructure & internet connectivity and behavioural intention (ß = 0.47; *p* < 0.05, R^2^ = 0.35). While perceived usefulness (ß = 0.15; *p* > 0.05) and security and privacy (ß = 0.03; *p* > 0.05) have an inverse relationship with behavioural intention. In addition, Table 5 shows the hypotheses testing results for the moderation effect derived from the findings of this study, where four models were tested. When ease of use is controlled, Model 2 shows that gender, age, education, ICT experience, and monthly income (Δ χ^2^/df = 5.32/1; > 3.84) have found to be significantly moderating the relationship between perceived ease of use → behavioural intention (ß = 0.39; *p* < 0.05, R^2^ = 0.47), perceived social influence → behavioural intention (ß = 0.23; *p* < 0.05, R^2^ = 0.47), as well as ICT infrastructure & internet connectivity → behavioural intention (ß = 0.53; *p* < 0.05, R^2^ = 0.47). When social influence is controlled, Model 3 shows that gender, age, education, ICT experience, and monthly income (Δ χ^2^/df = 2.45/1; < 3.84) have no moderating effect on the relationship between perceived ease of use → behavioural intention (ß = 0.39; *p* > 0.05, R^2^ = 0.47), social influence → behavioural intention (ß = 0.23; *p* > 0.05, R^2^ = 0.47), as well as ICT infrastructure & internet connectivity → behavioural intention (ß = 0.53, *p* > 0.05, R^2^ = 0.47). When ICT infrastructure & internet connectivity is controlled, Model 4 shows that gender, age, education, ICT experience, and monthly income (Δ χ^2^/df = 1.91/1; < 3.84) have no moderating effect on the relationship between perceived ease of use → behavioural intention (ß = 0.39; *p* > 0.05, R^2^ = 0.47), social influence → behavioural intention (ß = 0.23; *p* > 0.05, R^2^ = 0.47), as well as ICT infrastructure & internet connectivity → behavioural intention (ß = 0.53; *p* > 0.05, R^2^ = 0.47). Finally, when ease of use, social influence, and ICT infrastructure & internet connectivity are controlled, Model 5 shows that gender, age, education, ICT experience, and monthly income (Δ χ^2^/df = 13.25/3; > 3.84) have found to be significantly moderating the relationship between perceived ease of use → behavioural intention (ß = 0.39; *p* < 0.05, R^2^ = 0.47), perceived social influence → behavioural intention (ß = 0.23; *p* < 0.05, R^2^ = 0.47), as well as ICT infrastructure & internet connectivity → behavioural intention (ß = 0.53; *p* < 0.05, R^2^ = 0.47). Moreover, it is obvious from Fig. 3 that gender, age, education, ICT experience, and monthly income have a strong relationship with ICT infrastructure & internet connectivity (ß = 0.53), followed by their relationship with perceived ease of use (ß = 0.39), and then their relationship with social influence (ß = 0.23). Table 6 shows the summary of hypotheses testing.

**Table 5:** Overall analysis.

		Model 1	Model 2	Model 3	Model 4	Model 5
χ^2^		203.477				
df		289				
CFI		0.779				
NFI		0.627				
TLI		0.735				
RMSEA		0.053				
R^2^		0.35				
Perceived ease of use -> Intention		0.38*				
Social influence -> Intention		0.21*				
ICT -> Intention		0.47*				
Perceived ease of use × Gender × Age × Education × ICT experience × Monthly income						
χ^2^			269.329			
df			289			
∆χ^2^/df			5.32/1			
CFI			0.807			
NFI			0.680			
TLI			0.702			
RMSEA			0.052			
R^2^			0.47			
Perceived ease of use -> Intention			0.39*			
Social influence -> Intention			0.23*			
ICT -> Intention			0.53*			
Social influence × Gender × Age × Education × ICT experience × Monthly income						
χ^2^				279.743		
df				289		
∆χ^2^/df				2.45/1		
CFI				0.746		
NFI				0.623		
TLI				0.608		
RMSEA				0.059		
R^2^				0.47		
Perceived ease of use -> Intention				0.39*		
Social influence -> Intention				0.23*		
ICT -> Intention				0.53*		
ICT infrastructure × Gender × Age × Education × ICT experience × Monthly income						
χ^2^					267.255	
df					289	
∆χ^2^/df					1.91/1	
CFI					0.773	
NFI					0.671	
TLI					0.649	
RMSEA					0.062	
R^2^					0.47	
Perceived ease of use -> Intention					0.39*	
Social influence -> Intention					0.23*	
ICT -> Intention					0.53*	
Perceived ease of use × Gender × Age × Education × ICT experience × Monthly income Social influence × Gender × Age × Education × ICT experience × Monthly income						
ICT infrastructure × Gender × Age × Education × ICT experience × Monthly income						
χ^2^						288.458
df						289
∆χ^2^/df						13.25/3
CFI						0.768
NFI						0.631
TLI						0.608
RMSEA						0.053
R^2^						0.47
Perceived ease of use -> Intention						0.39*
Social influence -> Intention						0.23*
ICT -> Intention modration			Yes	No	No	0.53* Yes

**Note:**

* Significant at *p* < 0.05

**Table 6:** Summary of hypotheses testing.

Hypotheses	Results
HC1: Perceived usefulness has a positive relationship with Jordanian users’ intention to adopt smart city technologies.	Not supported
HC2: Perceived ease of use has a positive relationship with Jordanian users’ intention to adopt smart city technologies.	Supported
HC3: Security and privacy has a positive relationship with Jordanian users’ intention to adopt smart city technologies.	Not supported
HC4: Social influence has a positive relationship with Jordanian users’ intention to adopt smart city technologies.	Supported
HC5: ICT infrastructure & internet connectivity has a positive relationship with Jordanian users’ intention to adopt smart city technologies.	Supported
HC1a: Gender, Age, ICT experience, education and household income have significantly moderate the relationship between perceived usefulness and Jordanian users’ intention to adopt smart city technologies.	Not supported
HC2a: Gender, Age, ICT experience, education and household income have significantly moderate the relationship between perceived ease of use and Jordanian users’ intention to adopt smart city technologies.	Supported
HC3a: Gender, age, ICT experience, education and household income have significantly moderated the relationship between security and privacy and Jordanian users’ intention to adopt smart city technologies.	Not supported
HC4a: Gender, age, ICT experience, education and household income have significantly moderate the relationship between social influence and Jordanian users’ intention to adopt smart city technologies.	Supported
HC5a: Gender, age, ICT experience, education and household income have significantly moderate the relationship between ICT infrastructure & internet connectivity and Jordanian users’ intention to adopt smart city technologies.	Supported

The findings (See Table 6) specified that age, gender, education, ICT experience and monthly income have a robust impact on perceived ease of use, social influence, and ICT infrastructure & internet connectivity since R^2^ increases from 0.35 to 0.47. However, when these results come to the rigorousness of the relationship, demographic variables have more impact on ICT infrastructure & internet connectivity than perceived ease of use and social influence, as demonstrated in Fig. 3.

## Discussion

This article sought to find out the degree of citizens’ adoption and acceptance of the use of smart technologies in Jordan. Smart cities are those that use communication technology to make information-based decisions, improve the quality of life and increase work networks and services to maximize the benefit of their residents (Habib, Alsmadi & Prybutok, 2020). Smart cities run efficient systems and prepare advanced, as they seek to facilitate the residents’ communication with government agencies, where the population’s access to government services is easy and fast, education is better, humanitarian initiatives are effective, and relations between residents are at a satisfactory level (Ullah & Al-Turjman, 2021). Also, in the event of an interruption of services, the response of the emergency services is very quick to restore them to what they were (Perri, Giglio & Corvello, 2020).

The results showed that perceived ease of use has a positive relationship with Jordanian users’ intention to adopt smart city technologies. This result is consistent with (Gunabalan, Ooi & Yeap, 2022; Wang et al., 2022; Najdawi & Said, 2021; Yoo, Suh & Kim, 2020). As perceived ease of use is an essential construct in TAM, as it increases the acceptance and an ideal use of smart city technologies (Tavitiyaman, Zhang & Tsang, 2022). In this regard, Dirsehan & van Zoonen (2022) explained that perceived ease of use will improve understanding and dealing with a new system, and thus add a sense of satisfaction to citizens, who will not find complications that may hinder them from performing their daily activities, which will reflect positively on achieving a benefit from the system used. Therefore, smart cities have to enhance investments in existing data centers or build new ones, using concepts and technologies such as modular data centers, convergence infrastructures and software-defined technologies, in order to increase scalability and enhance standards and efficiency (Zhang & Liu, 2022). Also, institutional readiness, including the absence of legal and regulatory obstacles, is necessary to implement smart city initiatives in a smooth manner, through awareness campaigns that contribute to introducing users to smarter technologies (Wang et al., 2022).

Also, the results showed that ICT infrastructure & internet connectivity had the greatest impact on the Jordanian users’ intent and adoption of smart city technologies. This result is agree with (El Barachi et al., 2022; Haque, Bhushan & Dhiman, 2022; Kaluarachchi, 2022; Lim, Cho & Kim, 2021). As ICT infrastructure & internet connectivity is the main key in adopting the smart city model, since smart city initiatives are based on critical cloud computing infrastructure services and components. In this regard, Jordan seeks to digitize its economy by 2025 through The Royal Initiative REACH 2025, which was carried out through activities from the Ministry of Digital Economy and Entrepreneurship, as the idea of digitization revolves around transforming the traditional economy into a smart economy capable of solving problems and performing routine procedures that save time and effort (El Barachi et al., 2022). Where individuals, sectors and companies will be able to develop their businesses, which will reflect positively on the national economy. The citizens of Jordan, especially Amman, are the best example of the interaction of the smart citizen with technology. According to recent studies, Jordan is among the top countries in the world with an Internet penetration rate, where internet users are rising significantly in Jordan, reaching 6.84 million in 2021 (Al-Adwan et al., 2022).

The results showed that perceived usefulness has a negative relationship with Jordanian users’ intention to adopt smart city technologies. This result is inconsistent with Marimuthu, D’Souza & Shukla, (2022), Najdawi & Said, (2021), Manfreda, Ljubi & Groznik, (2021). As perceived usefulness can be used to determine the extent to which users accept or reject the adoption of smart cities, measure their services and quality, the intentions of their users, the expected benefit from this use, and the ease of access to information sources available on the Internet (Jnr & Petersen, 2022). As it is one of the honest and reliable variable for interpreting the acceptance of information systems (Manfreda, Ljubi & Groznik, 2021). However, perceived usefulness does not provide sufficient understanding for technology designers of the needs of technology beneficiaries to create an appropriate environment for technology acceptance (Martín-García, Redolat & Pinazo-Hernandis, 2022).

The results showed that security and privacy has a negative relationship with Jordanian users’ intention to adopt smart city technologies. This result contradicts (Ismagilova et al., 2022; Al-Turjman, Zahmatkesh & Shahroze, 2022; Neupane et al., 2021; Habib, Alsmadi & Prybutok, 2020), which explained that privacy and security are a key factor for the success of the smart city idea, especially with the increasing prevalence of smart solutions of all kinds, where many different parties participate in building the components of different smart solutions platforms. Despite the valuable proposition of cloud computing related to operational efficiencies, throughput, scalability and costs, challenges around data security, sensors and data portability remain a major source of concern in smart cities, especially when the data includes mission-critical information at the sector level or citizen information (Hassan et al., 2021). Therefore, governments and executives need to think carefully about their current cyber strategy and system and the risks involved, to be able to understand the challenges, and define their role in building a secure electronic environment for their communities, partners and governments (Al-Turjman, Zahmatkesh & Shahroze, 2022).

The results concluded that social influence has a positive relationship with Jordanian users’ intention to adopt smart city technologies. This result is agree with Gumz et al., (2022), Grandhi, Grandhi & Wibowo, (2021), that emphasized that social influence has a large impact on developed or smart cities in the first place, aiming to improve the level of health care and education, as well as the participation of communities in making developmental decisions that affect their lives, as the conditions of contemporary technology imposed a lot of new requirements on various social environments, perhaps the most prominent of which are urban environments, whose impact on technological manifestations has become clear, which made them have a high desire to accept all aspects of technological urbanization in a way that makes them an environment capable of development and cultural integration. It is intended for urban citizens to be more aware, creative and inclusive of all the city’s variables and infrastructure to achieve the principle of empowerment, active participation and maximum benefit from the available city services to ensure their right to participate in decision-making (Grandhi, Grandhi & Wibowo, 2021). The building of the smart citizen depends on an important element, which is knowledge, where attention is paid to building a society based on the management of urban life, where the educational experience is delivered in a homogeneous manner to all regions, whether urban or rural. A better and more prestigious educational level is also being relied upon for all citizens by adopting e-learning or cooperative education (Gumz et al., 2022). On the other hand, the huge and irregular urban expansion contributed to a jump in the rate of urbanization in Jordan, which led to a decline in the standard of living and the emergence of the problem of housing and a boring life. These factors lead to the expulsion of the population from the countryside and a high concentration in the cities, especially the major ones. This is in addition to the existence of many problems represented by the high prices of land, the spread of backward neighborhoods, and the extension of cities outside their borders (El Barachi et al., 2022).

The results show that gender, age, education, ICT experience, and monthly income have found to be significantly moderating the relationship between perceived ease of use and behavioural intention, perceived social influence and behavioural intention, as well as ICT infrastructure & internet connectivity and behavioural intention. Alderete (2021) found that citizens’ acceptance of smart city technology is directly dependent on demographic factors, especially with regard to education level, access to and use of ICTs. But this result disagrees with Yeh (2017), who found that demographic factors do not influence citizens’ attitudes toward smart city technologies with respect to gender, age, and education. This results may be attributed to the disparity between the educational level of citizens and their use of smart devices to interact with the smart city, which confirms the importance of demographic factors in the success or failure of smart city initiatives, in addition to the necessity of providing digital infrastructure to support communications and link remote areas with digitally qualified areas (Hou et al., 2020).

Information and communication technology is the main driver of smart city initiatives, as it relies on smart computing technologies applied to critical infrastructure services and components (El Barachi et al., 2022). In Jordan, there are a group of smart projects that depend on technological inevitability, aiming to stimulate the growth of its private sector, increase the efficiency and sustainability of its services, improve its investment attractiveness, and enhance its global competitiveness. This is due to the expansion of the use of technical solutions for information and communication systems in various aspects of life, in a way that contributes to facilitating the daily life of the population, raising the level of quality of services in various sectors, rationalizing the consumption of available resources, and facilitating the exchange of data and information. Nevertheless, the smart city has its own concept, main and secondary dimensions, and indicators, which combine digital communication technologies and urban development on the one hand, and the goals of sustainable development on the other (Sharma et al., 2020) In this regard, Jordan is witnessing major transformations to take advantage of smart technology in order to achieve sustainable development (Fernandez-Anez et al., 2020). For example, to address the environmental challenges associated with overcrowding in cities, the solution lies in providing innovative solutions to improve the quality of life, protect the shares of natural resources for future generations, and meet their environmental needs. To sum up, the issue of consensus on setting broad lines for adopting the idea of smart cities in Jordan necessitates research and standing on its current situation and the development of its various sectors such as the environment, tourism, economy, and others. The current infrastructure must be assessed, an action plan consisting of phases is being prepared, and the availability of financial and other resources necessary for its implementation must be verified.

## Conclusion and Implication

This study is the first attempt in the application of TAM and UTAUT as a basis models for understanding the factors that contribute to predicting or affecting the behavioural intention of citizens to adopt smart city technologies, hence, this study is expected to contribute to literature through the introduction into Jordanian context. The theoretical contribution also comes from being a reason to clarify the current situation in Jordanian citizens’ acceptance of smart city technologies and the extent to which they are expected to be optimally used, which enriches knowledge and science in this field. On the other hand, the practical contribution lies in understanding the customs and environment of Jordanian citizens as it is an important factor in determining the user’s behavior towards the used smart city technologies and the extent of their acceptance of learning and the accumulation of their experiences or their reluctance to do so, or the preservation of the traditional methods that citizens are accustomed to. Moreover, the practical contribution appears in the integration of TAM and UTAUT, which contains many factors that decision-makers in Jordan must understand, and anticipate the behaviour of citizens regarding them.

For Jordan, adopting smart cities is the best investment for the future, especially since its benefits are many and puts Jordan in the ranks of developed countries. One of the most important practical implications of adopting smart city technologies in Jordan is that all information is available in an automated way, and use of all data and take decisions through safe and easy programs and robots to serve all sectors, whether economic, health, educational, scientific, transportation and others. Information and communication technology infrastructure is a prerequisite for the success of smart cities and the effectiveness of their services. In order for the many systems in smart cities to work, integrate and harmonize with each other, a specific set of standards must be strictly adhered to. Moreover, the successful implementation of the idea of smart cities in Jordan will contribute to economic growth, prosperity, global competitiveness, improving innovation rates, providing better and faster services, in addition to transparency, and creating great opportunities for various sectors. Furthermore, this study will contribute to supporting decision-makers in formulating solid outputs that lead a global revolution in the green economy, rebuilding, using renewable energy, developing new lifestyles, and benefiting from the technological revolution in important sectors such as transportation and communications, which contributes to reducing harmful emissions into the environment. Also, this study seeks to developing advanced future visions that meet the requirements of future citizens, and provide advanced digital infrastructure in Jordan.

Regardless of the problems that Jordan is still grappling with, even the smart ones, the studies unanimously agree that the current trend towards smart cities is the first feature of the transformation towards the cities of the future. We cannot imagine our current cities steadfast in the face of terrible digital development, the spread of the Internet, and the virtual world. However, the process of planning and designing smart cities is closely related to social sustainability, which is represented in the ability to change the habits, traditions and behavior of the residents or users. Therefore, it was necessary to ensure the availability of an appropriate amount of interaction within the residential neighborhoods so that communication turns into a fruitful and harmonious shared life. This maintains social, cultural and urban sustainability in Jordan, and makes community life more cohesive.

Social change is in itself difficult to achieve, as it requires improving the level of health care and education, as well as the participation of communities in making developmental decisions that affect their lives. Since the population of Jordan varies in the level of income between limited, middle, above average, and affluent, the design of sustainable smart cities requires the provision of public and basic services at a cost consistent with the income of each segment, such as housing, food, water, electricity, and fuel services. The aesthetics and cleanliness of public gardens, parks, roads and sidewalks are among the most important factors for the residents’ happiness and satisfaction. Furthermore, the advancement of residential neighborhoods in Jordan to create a positive interactive social life among the population is one of the main goals. Therefore, a series of periodic housing seminars must be organized with the participation of experts and specialists from different countries of the world, to exchange information, experiences and practices, in a way that represents a special form of social support and achieves individual benefits and a sense of community.

### Limitations and future research

Concerning limitations, this research adopted quantitative method to test the research hypotheses, as among the shortcomings of this method is that the errors of inspection and measurement, which the researchers may fall into. Despite this limitation, it can be used as an opportunity to describe the need for future research to complement what the current study left off by following a different methodology. Furthermore, TAM and UTUAT was used to explore and investigate Jordanian citizens decisions to adopt and accept smart cities technologies, thus, it is possible that future research will focus on using other models and theories in Jordanian context, such as diffusion of innovation (DOI) theory and the theory of reasoned action (TRA). Moreover, the lack of extensive data in Jordanian context is another limitation, as the relatively limited data is a major hindrance to this study given the significance of such data in conducting the research. Thus, future research could include other geographical area for an appropriate understanding of smart cities adoption and acceptance.

## Supplemental Information

10.7717/peerj-cs.1289/supp-1 Supplemental Information 1 Raw Data.

10.7717/peerj-cs.1289/supp-2 Supplemental Information 2 Survey.

10.7717/peerj-cs.1289/supp-3 Supplemental Information 3 Original language questionnaire.

## Additional Information and Declarations

### Data Availability

The following information was supplied regarding data availability: The raw data and questionnaire are available in the Supplemental Files.
