# Supplementary material for: Investigating smart city adoption from the citizen’s insights: empirical evidence from the Jordan context
Source: PeerJ Comput Sci. 2023 Mar 20;9:e1289. doi: 10.7717/peerj-cs.1289 (PMC10280567; doi:10.7717/peerj-cs.1289)
Supplement: Supplemental Information 2 [file peerj-cs-09-1289-s002.pdf]

Clarification: In this research, we study the factors that may hinder the development of the capital, Amman, as a smart city, with all its facilities that are based on the Internet and information technology, including government services, educational services, socail services and health facilities.

Smart City Concept: According to the International Telecommunication Union, smart city is an innovative city that uses information and communication technology to improve the quality of life, the efficiency of urban operations and services, and the ability to compete, while at the same time meeting the needs of current and future generations in terms of economic, social, environmental, and cultural aspects. It can be said that they are cities that use digital maps that provide real-time information on the status of specific neighborhoods, institutions and complexes, in addition to the city's air, sea and land space, in addition to automating many manual transactions and converting them into electronic ones that can be implemented through smart devices such as phones and computers. By making all this information available in real time to managers, police, citizens and government educational, service and health departments, anyone can complete many transactions electronically or know the presence of buses, congestion areas, energy consumption, air quality and other services, in addition to facilitating the work of health services, grocers and stores owners, marketing experts and educators, social workers and many other employees in the performance of their work based on real-time information. Google Maps application can be considered a simple example of the applications that will be available in the smart city.

Commitment: The information and responses collected are completely private and confidntail. We do not share these individual responses with anyone and they are used only for purposes related to presenting and improving scientific research.

Note: we removed all corresponding info for us, which disclose our personal info and emails that indicate to our self. As we provided you with a copy for Editors and/or reviewers' purposes.

Demographics’ Profiles

1. **Gender:**
   a) Female
   b) Male

2. **Age**
   a) 18 – 25
   b) 26 – 35
   c) 36 – 45
   d) 46 – 50
   e) Above 50

3. **Highest Education Level**
   a) Secondory school or less
   b) Diploma
   c) Bacheolrs
   d) Graduate Studies

4. **Information and communication technology (ICT) experience**
   a) Weak
   b) Acceptable
   c) Good
   d) Very Good
   e) Excellent

5. **The monthly income**
   a) Less than 500 JOD
   b) 500 – 700 JOD
   c) 700 – 1000 JOD
   d) Above 1000 JOD

Amman's Smart City Adoption and acceptance

1. **Perceived Usefulness and Enjoyment**

Question	Strongly Disagree	Disagree	Neutral	Agree	Strongly Agree
Amman smart city increases my productivity.					
Amman smart city allows me to improve my work performance and quality.					
Amman smart city saves me time.					
Overall, I would find Amman smart city to be advantageous.					

2. **Perceived Ease of Use**

Question	Strongly Disagree	Disagree	Neutral	Agree	Strongly Agree
My interaction with smart city is clear and understandable.					
Learning to operate smart city and following the guidance is easy to me.					
It is easy and quick for me to become skillful at using smart city.					
I think smart city will be user friendly and easy to use.					
Overall, I think the smart city is easy to get to do what I wanted to do.					

3. **Security and privacy**

Question	Strongly Disagree	Disagree	Neutral	Agree	Strongly Agree
I think my information is not disclosed to unwanted authorities or personals.					
I think my confidentialities of information remains protected.					
I think the security aspect of the IT-enabled system is not compromised under any circumstances.					

Users are adequately trained and aware of how to use the IT enabled services safely and securely.					
Overall, I would find the IT-enabled services of smart city are having a high degree of security features which can keep the digital services fully secured.					

4. ICT Infrastructure and Inadequate Internet connectivity

Question	Strongly Disagree	Disagree	Neutral	Agree	Strongly Agree
I think the smart city services are efficient and user friendly to the residents of smart city.					
I think the functionalities will be adequately designed to meet the needs of the users with full satisfaction.					
I think the smart city systems are well maintained providing good-quality services to the users.					
I think the information will be continuously updated with latest information in place.					
Overall, I think the system is reliable and it maintains the performance as per the requirements.					

5. Social Influence

Question	Strongly Disagree	Disagree	Neutral	Agree	Strongly Agree
I think that friends and family members will influence my decision to use smart city.					
Mass media (e.g.: TV, radio and newspaper) will influence my decisions in using smart city.					
Overall, I will use smart city if my colleagues use it.					

6. **Behavioral Intention**

Question	Strongly Disagree	Disagree	Neutral	Agree	Strongly Agree
I will use the smart city once adopted.					
I will purchase smart city enabled phones once adopted,					
I predict that I will continue to use the smart city applications on a regular basis.					
