# Supplementary material for: Investigating smart city adoption from the citizen’s insights: empirical evidence from the Jordan context
Source: PeerJ Comput Sci. 2023 Mar 20;9:e1289. doi: 10.7717/peerj-cs.1289 (PMC10280567; doi:10.7717/peerj-cs.1289)
Supplement: Supplemental Information 3 [file peerj-cs-09-1289-s003.pdf]

عمّان كمدينة ذكية

The survey will take approximately 4 minutes to complete.

توضيح: نقوم في هذا البحث على دراسة العوامل التي قد تعيق تطوير العاصمة عمّان كمدينة ذكية قائمة كل مرافقها على الانترنت وتكنولوجيا المعلومات بما في ذلك المنشآت الحكومية والتعليمية والخدماتية والصحية.

مفهوم المدينة الذكية: وفقاً للاتحاد الدولي للاتصالات هي مدينة مبتكرة تستخدم تكنولوجيا المعلومات والاتصالات لتحسين نوعية الحياة، وكفاءة العمليات والخدمات الحضرية، والقدرة على المنافسة، وتلبي في الوقت ذاته احتياجات الأجيال الحالية والقادمة فيما يتعلق بالجوانب الاقتصادية والاجتماعية والبيئية، والثقافية.
ويمكن القول أنها مدن تستخدم خرائط رقمية توفر معلومات في الوقت الحقيقي عن حالة أحياء ومؤسسات ومجمعات محددة، إضافة إلى المجال الجوي والبحري والبري للمدينة، إضافة إلى أتمتة الكثير من المعاملات اليدوية وتحويلها إلى الكترونية يمكن تنفيذها من خلال الأجهزة الذكية كالهواتف والحواسيب.. وبإتاحة كل هذه المعلومات في وقتها الحقيقي للمديرين والشرطة والمواطنين والدوائر الحكومية التعليمية والخدماتية والصحية، يمكن لأي شخص إنجاز الكثير من المعاملات الكترونياً أو معرفة وجود الحافلات ومناطق الازدحام واستهلاك الطاقة وجودة الهواء وغيرها من الخدمات، إضافة إلى تسهيل أعمال الخدمات الصحية وأصحاب البقالة والمتاجر وخبراء التسويق والمُعلمين والأخصائيين الاجتماعيين وغيرهم الكثير من الموظفين في آداء أعمالهم بناء على معلومات لحظية.. ويمكن اعتبار تطبيق خرائط جوجل مثال بسيط على التطبيقات التي ستكون متاحة في المدينة الذكية.

تعهد: المعلومات والردود التي يتم جمعها خاصة تمامًا. نحن لا نشارك هذه الردود الفردية مع أي جهة ويتم استخدامها فقط للأغراض المتعلقة بتقديم وتحسين البحث العلمي.

القائمون على البحث:
د. منير نصير، جامعة الأمير سطام بن عبدالعزيز
د. محمد الشرعة، جامعة آل البيت

Personal Information: المعلومات الشخصية

1. **Gender: الجنس**

- ◯ أنثى
- ◯ ذكر

2. **Age: العمر**

- 18-25
- 26-35
- 36-45
- 46-50
- أكبر من 50 سنة

3. **Highest education level: المستوى الدراسي**

- ثانوية عامة فما دون
- دبلوم
- بكالوريوس
- دراسات عليا
- Other

4. **information and communication technology (ICT) experience: الخبرة في استخدام تكنولوجيا المعلومات**

- ضعيف
- مقبول
- جيد
- جيد جداً
- ممتاز

5. **The monthly income: الدخل الشهري**

- أقل من 500 دينار
- 500-700 دينار
- 700-1000 دينار
- أكثر من 1000 دينار

Amman Smart City: عمّان كمدينة ذكية

6. Perceived Usefulness and Enjoyment الفائدة المرجوة من المدينة الذكية

	Strongly Disagree بشدة لا أتفق	Disagree لا أتفق	Neutral محايد	Agree أتفق	Strongly Agree أتفق بشدة
Amman smart city increases my productivity ترفع المدينة الذكية في عمّان من قدرتي الانتاجية	○	○	○	○	○
Amman smart city allows me to improve my work performance and quality. تتيح لي المدينة الذكية في عمّان تطوير آداء وجودة عملي	○	○	○	○	○
Amman smart city saves me time. تسهم المدينة الذكية في عمّان في الحفاظ على وقتي	○	○	○	○	○
Overall, I would find Amman smart city to be advantageous. بشكل عام [illegible]	○	○	○	○	○

Strongly Disagree بشدة لا أتفق | Disagree لا أتفق | Neutral محايد | Agree أتفق | Strongly Agree أتفق بشدة

ارى ان تطوير عمّان كمدينة ذكية سيكون مفيداً.

7. Perceived Ease of Use سهولة استخدام المدينة الذكية

	Strongly Disagree بشدة لا أتفق	Disagree لا أتفق	Neutral محايد	Agree أتفق	Strongly Agree أتفق بشدة
My interaction with smart city is clear and understandable. تفاعلي مع المدينة الذكية سيكون محدداً ومفهوماً وواضحاً	○	○	○	○	○
Learning to operate smart city and following the guidance is easy to me. تعلم استخدام المدينة الذكية واتباع تعليماتها سيكون سهلاً بالنسبة لي	○	○	○	○	○
It is easy and quick for me to become skillful at using smart city. أعتقد سأستطيع امتلاك مهارات التعامل مع المدينة الذكية بسرعة وسهولة.	○	○	○	○	○

Strongly Disagree بشدة لا أتفق | Disagree لا أتفق | Neutral محايد | Agree أتفق | Strongly Agree أتفق بشدة

I think smart city will be user friendly and easy to use. أعتقد أن المدينة الذكية ستكون صديقة وسهلة الاستخدام بالنسبة للمستخدمين.	○	○	○	○	○
Overall, I think the smart city is easy to get to do what I wanted to do. بشكل عام أعتقد أن المدينة الذكية ستعمل على تسهيل تنفيذ الأعمال التي أريد القيام بها.	○	○	○	○	○

8. Security and privacy أمان وسرية المستخدمين

	Strongly Disagree بشدة لا أتفق	Disagree لا أتفق	Neutral محايد	Agree أتفق	Strongly Agree أتفق بشدة
I think my information is not disclosed to unwanted authorities or personals. أعتقد أن بياناتي التي أستخدمها ضمن المدينة الذكية لن تكون متاحة لأي سلطات أو جهات لا أرغب باطلاعهم	○	○	○	○	○
	Strongly Disagree بشدة لا أتفق	Disagree لا أتفق	Neutral محايد	Agree أتفق	Strongly Agree أتفق بشدة

عليها.

	Strongly Disagree بشدة لا أتفق	Disagree لا أتفق	Neutral محايد	Agree أتفق	Strongly Agree أتفق بشدة
I think my confidentialities of information remains protected. أعتقد أن سرية المعلومات الخاصة بي تبقى محمية.	○	○	○	○	○
I think the security aspect of the IT-enabled system is not compromised under any circumstances. أعتقد أن جانب السرية للمدينة الذكية لن يتم المساس به تحت أي ظروف.	○	○	○	○	○
Users are adequately trained and aware of how to use the IT enabled services safely and securely. أعتقد أنه سيتم تدريب المواطنين بشكل كافي بكيفية استخدام خدمات المدينة الذكية بشكل آمن وسري.	○	○	○	○	○

Overall, I would find the IT-enabled services of

	Strongly Disagree بشدة لا أتفق	Disagree لا أتفق	Neutral محايد	Agree أتفق	Strongly Agree أتفق بشدة

smart city are having a high degree of security features which can keep the digital services fully secured. بشكل عام، أعتقد أن خدمات المدينة الذكية سوف تتمتع بدرجة عالية من الأمان والسرية التي تضمن لي خدمة سرية وآمنة بالكامل.	○	○	○	○	○

9. ICT Infrastructure and Inadequate Internet connectivity البنية التحتية

	Strongly Disagree بشدة لا أتفق	Disagree لا أتفق	Neutral محايد	Agree أتفق	Strongly Agree أتفق بشدة
I think the smart city services are efficient and user friendly to the residents of smart city. أعتقد أن خدمات المدينة الذكية في عمّان سوف تتسم بالكفاءة وسهولة الاستخدام لسكان المدينة الذكية.	○	○	○	○	○
I think the functionalitie s will be	Strongly Disagree بشدة لا أتفق	Disagree لا أتفق	Neutral محايد	Agree أتفق	Strongly Agree أتفق بشدة

adequately designed to meet the needs of the users with full satisfaction. أعتقد أن وظائف المدينة الذكية سيتم تصميمها بشكل يلبي متطلبات المواطنين وينال رضاهم.	○	○	○	○	○
I think the smart city systems are well maintained providing good-quality services to the users. أعتقد أن أنظمة المدينة الذكية ستحافظ على استمرارية توفير خدمة عالية الجودة للمواطنين.	○	○	○	○	○
I think the information will be continuously updated with latest information in place. أعتقد أن معلومات المدينة الذكية سيتم تحديثها باستمرار بآخر المعلومات على أرض الواقع.	○	○	○	○	○
Overall, I think the system is reliable and it maintains the	Strongly Disagree بشدة لا أتفق	Disagree لا أتفق	Neutral محايد	Agree أتفق	Strongly Agree أتفق بشدة

performance as per the requirements. بشكل عام، أعتقد أن المدينة الذكية ستكون موثوقة وتحافظ على آداءها وفق المتطلبات.

10. Social Influence التأثير الاجتماعي

	Strongly Disagree بشدة لا أتفق	Disagree لا أتفق	Neutral محايد	Agree أتفق	Strongly Agree أتفق بشدة
I think that friends and family members will influence my decision to use smart city. أعتقد أن الأصدقاء وأفراد العائلة سيؤثرون على قراري باستخدام المدينة الذكية.	○	○	○	○	○
Mass media (e.g.: TV, radio and newspaper) will influence my decisions in using smart city. ستؤثر وسائل الإعلام (مثل التلفزيون والراديو والصحف) على قراراتي في استخدام المدينة الذكية.	○	○	○	○	○
Overall, I will use smart city if my colleagues use it. بشكل عام ، سأستخدم المدينة الذكية إذا استخدمها زملائي.	○	○	○	○	○

11. Behavioral Intention احتمالية استخدام المدينة الذكية

	Strongly Disagree بشدة لا أتفق	Disagree لا أتفق	Neutral محايد	Agree أتفق	Strongly Agree أتفق بشدة
I will use the smart city once adopted. سأستخدم المدينة الذكية بمجرد اعتمادها.	○	○	○	○	○
I will purchase smart city enabled phones once adopted, سأشتري الهواتف التي تدعم المدينة الذكية بمجرد تطبيقها،	○	○	○	○	○
I predict that I will continue to use the smart city applications on a regular basis. أتوقع أنني سأستمر في استخدام تطبيقات المدينة الذكية بشكل منتظم.	○	○	○	○	○

12. شكراً جزيلاً على مساهمتك في هذا الجهد العلمي، يمكنك هنا إضافة أي تعليق أو ملاحظة

This content is neither created nor endorsed by Microsoft. The data you submit will be sent to the form owner.

Microsoft Forms
